# Magnetic Shielding Analysis of Bonding in [1.1.1]Propellane

**DOI:** 10.1021/acs.jpca.2c06450

**Published:** 2023-01-19

**Authors:** Peter B. Karadakov, Ben Stewart, David L. Cooper

**Affiliations:** † Department of Chemistry, University of York, Heslington, YorkYO10 5DD, U.K.; ‡ Department of Chemistry, University of Liverpool, LiverpoolL69 7ZD, U.K.

## Abstract

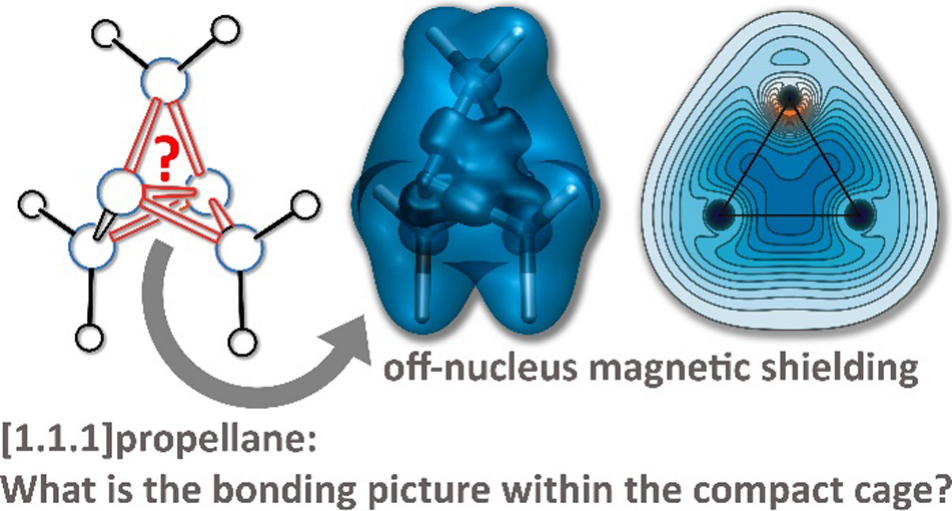

The bonding in [1.1.1]propellane, bicyclo[1.1.0]butane, bicyclo[1.1.1]pentane, tetrahedrane, and cyclopropane is investigated by analyzing changes in the off-nucleus isotropic magnetic shielding within the space surrounding each of these molecules and, for [1.1.1]propellane, by examining also the diamagnetic and paramagnetic contributions to this shielding. Any shielding arising from the two “exo” sp^3^-like hybrid atomic orbitals on the bridgehead carbon atoms that have been used to support the idea of an inverted bond between these two atoms is found to be almost entirely contained within the [1.1.1]propellane cage and to contribute to a strongly shielded central region. This strongly shielded region suggests the establishment of a mainly covalent bonding interaction involving all carbon atoms that cannot be straightforwardly decomposed into contributions from individual carbon–carbon bonds. The emergence of the strongly shielding central region is traced by comparing the shielding variations in and around molecules with one three-membered carbon ring (cyclopropane), two fused three-membered carbon rings (bicyclo[1.1.0]butane), and three fused three-membered carbon rings ([1.1.1]propellane).

## Introduction

Bonding in [1.1.1]propellane (tricyclo[1.1.1.0^1,3^]pentane) and, in particular, the questions of whether there is a central bond connecting the two bridgehead carbon atoms and, if so, what is the nature of that bond, have been debated by theoretical chemists over many years.^1^ If it is present, a bond between the bridgehead carbon atoms would imply that [1.1.1]propellane incorporates three three-membered carbon rings fused along that bond. The prevalent view in the literature, mostly based on breathing-orbital valence bond (BOVB) calculations,^2^ is that this central bond does exist and that it is an example of a so-called charge-shift bond (CSB).^3^ In the BOVB description, the bond between the bridgehead carbon atoms is established with the participation of two “exo” sp^3^-like hybrid atomic orbitals (HAOs) pointing outward of the [1.1.1]propellane cage and, as a consequence, the bond is said to be “inverted.” In contrast to standard covalent bonds, its strength arises in BOVB calculations from resonance between covalent and ionic components, C··C ↔ (C^+^:C^–^ + C^–^:C^+^). The covalent component C··C, which involves the singlet-coupled electrons in the two HAOs, is thought not to be sufficiently “bonding” to overcome the large repulsions from the six “wing” C–C bonds, and hence the need to bring in the ionic components, which are also insufficient on their own to explain the bonding. Such views, and indeed the very existence of the inverted bond, are still actively being queried^4^ and defended robustly.^5^ Certainly, it has been shown that localization of the two active space orbitals in a CASSCF(2,2) wavefunction (complete active space self-consistent field with “2 electrons in 2 orbitals”) for [1.1.1]propellane leads to a description of the inverted bond that resembles closely the CSB model;^6^ one difference from the BOVB wavefunction is that the localized CASSCF(2,2) active space orbitals are orthogonal, which “pushes” them further out of the interior of the cage. Even so, this does mean that a wavefunction incorporating the essential features of the CSB model for the inverted bond in [1.1.1]propellane had been used to describe this molecule many years ago because the two-configuration SCF (TCSCF) wavefunction that was employed by Feller and Davidson^7^ is equivalent to a CASSCF(2,2) construction.

In this paper, we present a different interpretation of the bonding in [1.1.1]propellane. Instead of looking for individual carbon–carbon bonds and then trying to elucidate their nature and interactions, we analyze the overall bonding picture within the [1.1.1]propellane cage using a visual approach that involves the calculation of the off-nucleus isotropic magnetic shielding, σ_iso_(**r**), as a function of position in the space surrounding the molecule. The most popular example of an off-nucleus shielding in chemistry is the nucleus-independent chemical shift (NICS),^8^ an aromaticity criterion suggested by Schleyer and co-workers which, in its original definition, uses a single isotropic shielding calculated at the center of an aromatic or antiaromatic ring and taken with an inversed sign.^9^ The approach we use is closer in spirit to the work of Wolinski^10^ who analyzed the changes in the off-nucleus shielding tensor along the molecular axes of linear molecules and to the isotropic shielding isosurfaces investigated by Klod and Kleinpeter.^11^ An off-nucleus magnetic shielding tensor **σ**(**r**) can be calculated, in analogy to the nuclear magnetic shielding tensor, as a second-order response property.^10^
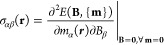
1where *E*(**B**, {**m**}) is the energy of the molecule in the presence of an external magnetic field **B**, {**m**} stands for the collection of magnetic moments of the nuclei and of suitable probes, say, neutrons,^10^ placed at **r** and at any other off-nucleus locations of interest, and α and β denote the Cartesian coordinates *x*, *y*, and *z*. Differentiating *E*(**B**, {**m**}) with respect to *m*_α_(**r**) first leads to the expression^12,13^
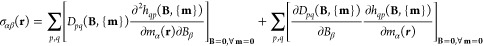
2where *D_pq_*(**B**, {**m**}) and *h_qp_*(**B**, {**m**}) are elements of the one-electron density matrix and of the one-electron part of the Hamiltonian, respectively, in terms of gauge-including atomic orbitals (GIAOs). The off-nucleus isotropic magnetic shielding corresponding to eq 2 is defined as σ_iso_(**r**) = 1/3[σ_*xx*_(**r**) + σ_*yy*_(**r**) + σ_*zz*_(**r**)]. The two terms in eq 2 can be assumed to correspond to the diamagnetic and paramagnetic contributions to the shielding tensor, **σ**(**r**) = **σ**^d^(**r**) + **σ**^p^(**r**); if use is made of the natural orbital connection,^14−16^ this assumption has been shown to work well for nuclear shieldings^16^ and it can be shown to work equally well for off-nucleus shieldings. The natural orbital connection ensures maximum similarity, in a least-squares sense, between the orthonormalized perturbed molecular orbitals (MOs) and the unperturbed (unmodified) MOs.^17^ This connection provides a suitable partitioning of the shielding tensors defined in terms of GIAOs into diamagnetic and paramagnetic contributions which coincide in the basis set limit with the usual definitions for perturbation-independent AOs.

According to eq 2, the diamagnetic contribution depends on the one-electron density matrix, and the paramagnetic contribution depends on the extent to which the elements of this density matrix can be perturbed by an external magnetic field. We note that the derivatives *∂D_pq_*(**B**, {**m**})/*∂B*_β_ are imaginary and, therefore, to first order, the one-electron density matrix does not change as a result of the perturbation. The electron density along a chemical bond, when exposed to an external magnetic field, shields the bond and this shielding persists even if the strength of the magnetic field approaches zero. The shielding along and around a bond can be examined by calculating off-nucleus shieldings and their diamagnetic and paramagnetic contributions at a number of points within the space surrounding the bond; the data at these points can be assembled into an isosurface or a contour plot. The balance between the two terms in eq 2 is such that the off-nucleus isotropic magnetic shielding usually increases and reaches a maximum near the midpoint of a bond, rendering most of the bond well-shielded, in contrast to electron density which quickly decreases away from atoms. Hence, off-nucleus isotropic shielding plots usually show higher levels of bond-specific details over the whole length of a chemical bond, which makes the differences between bonds easy to visualize.^18^ As a rule, the shielding over a bond increases with bond multiplicity and, in most cases, stronger bonds are more shielded than weaker bonds. For example, analysis of the changes in σ_iso_(**r**) has been used to demonstrate that the carbon–carbon bond in dicarbon, C_2_, is “bulkier,” which is consistent with higher multiplicity, but also weaker than the triple carbon–carbon bond in ethyne, C_2_H_2_.^19^ It is interesting in this context to note that C_2_ was of direct relevance to an earlier description of the bonding in [1.1.1]propellane in terms of three-center two-electron “σ-bridged π bonds,” arising from the interaction of the MOs on a C_2_ moiety and on the three methylene (CH_2_) fragments.^20^ In conjugated cyclic systems with higher-energy π electrons, such as cyclobutadiene, the balance between the two terms in eq 2 can change in favor of the negative second term above and below the ring, leading to the appearance of a distinctly deshielded dumbbell-shaped region which decreases shielding over bonds and can be associated with antiaromaticity.^21,22^ On the other hand, strongly shielded central regions have been observed in shielding calculations on singlet excited states of benzene^22^ and cyclooctatetraene.^23^

In order to understand better the bonding pattern established within the very tight space inside the compact propellane cage, we compare the isotropic magnetic shielding distribution around [1.1.1]propellane to those around bicyclo[1.1.1]pentane, in which the hydrogen atoms connected to each bridgehead carbon atom (C_b_) prevent the establishment of a C_b_–C_b_ bond, as well as to those around tetrahedrane, the hypothetical hydrocarbon featuring the smallest carbon cage, and around bicyclo[1.1.0]butane and cyclopropane, the smallest examples of molecules with two fused and one three-membered carbon rings, respectively. We also examine the spatial variations around [1.1.1]propellane in the diamagnetic and paramagnetic contributions to shielding, and in the total electron density and its Laplacian.

## Computational Details

The *D*_3*h*_ geometries of [1.1.1]propellane and bicyclo[1.1.1]pentane, the *C*_2*v*_ geometry of bicyclo[1.1.0]butane, and the *T_d_* geometry of tetrahedrane were optimized at the B3LYP-D3(BJ)/def2-TZVP level (B3LYP with Grimme’s D3 empirical dispersion corrections and Becke–Johnson damping, within the def2-TZVP basis set, as implemented in GAUSSIAN^24^). The *D*_3*h*_ geometry of [1.1.1]propellane and the *C*_2*v*_ geometry of bicyclo[1.1.0]butane were also optimized at the CASSCF(2,2)/def2-TZVP level using an active space analogous to that in the TCSCF wavefunction of Feller and Davidson.^7^ All optimized geometries were confirmed as local minima through harmonic frequency calculations. For cyclopropane, we used an experimental geometry derived from the analysis of its microwave spectrum.^25^

σ_iso_(**r**) volume data required for the construction of isosurfaces and contour plots were obtained by means of B3LYP-GIAO/6-311++G(d,p) calculations [B3LYP with gauge-including atomic orbitals, within the 6-311++G(d,p) basis set], at the B3LYP-D3(BJ)/def2-TZVP geometries of [1.1.1]propellane, bicyclo[1.1.0]butane, bicyclo[1.1.1]pentane and tetrahedrane, and at the experimental geometry of cyclopropane. Additional σ_iso_(**r**) volume data were obtained for [1.1.1]propellane and bicyclo[1.1.0]butane by means of CASSCF(2,2)-GIAO/6-311++G(d,p) calculations at the respective CASSCF(2,2)/def2-TZVP geometries. In all volume data calculations, σ_iso_(**r**) was evaluated on regular three-dimensional grids of points with a spacing of 0.05 Å. To reduce computational effort, shielding tensors were calculated for each grid at the symmetry-unique points (using Abelian symmetry only), and then, data were replicated by symmetry.

For visualization purposes, all σ_iso_(**r**) values from the B3LYP-GIAO/6-311++G(d,p) calculations on [1.1.1]propellane, bicyclo[1.1.0]butane, bicyclo[1.1.1]pentane, tetrahedrane, and cyclopropane, as well as from the CASSCF(2,2)-GIAO/6-311++G(d,p) calculations on [1.1.1]propellane and bicyclo[1.1.0]butane, were assembled in GAUSSIAN cube files.^26^ To enable comparisons of the isotropic nuclear shieldings for the molecules studied in this paper to those for ethane and ethene, B3LYP-GIAO/6-311++G(d,p) calculations for ethane and ethene were carried out at the experimental geometries obtained, respectively, from spectroscopic data^27,28^ and from a combination of rotational spectroscopy and quantum chemical calculations.^29^

To construct contour plots for [1.1.1]propellane of the total electron density (ρ) and of the Laplacian of the total electron density (∇^2^ρ), these two quantities were evaluated at the B3LYP/6-311++G(d,p) and CASSCF(2,2)/6-311++G(d,p) levels with the GAUSSIAN CUBEGEN utility program,^26^ using two-dimensional grids of points with a spacing of 0.05 Å in one of the σ_v_ symmetry planes.

All calculations reported in this paper were carried out in the gas phase and were performed using GAUSSIAN,^24^ except for the CASSCF(2,2)-GIAO calculations, which were performed using DALTON.^30^ All optimized geometries, additional computational details, and the GAUSSIAN cube files with shielding data are included in the Supporting Information.

## Results and Discussion

**Figure 1:**
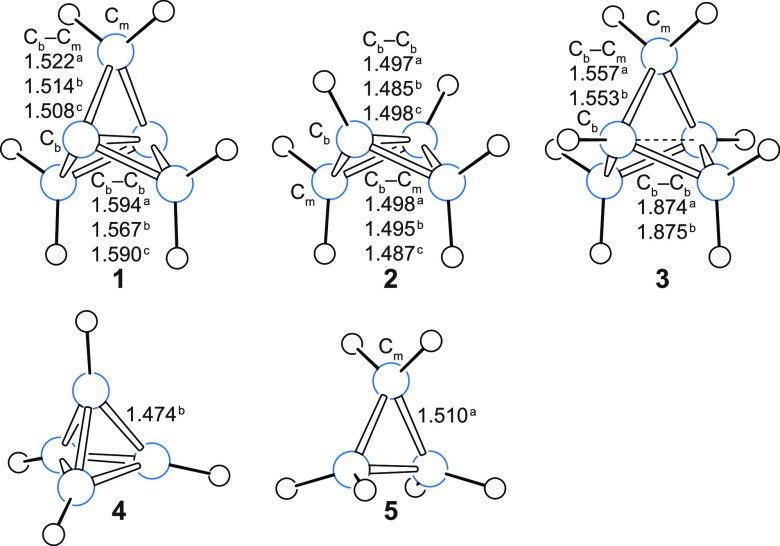
Geometries of [1.1.1]propellane **1**, bicyclo[1.1.0]butane **2**, bicyclo[1.1.1]pentane **3**, tetrahedrane **4,** and cyclopropane **5** with C–C distances (in Å) from ^a^experimental geometries (gas-phase electron diffraction for **1**(31) and **3**,^32^ microwave spectrum analysis for **2**(33) and **5**(25)), and from ^b^B3LYP-D3(BJ)/def2-TZVP and ^c^CASSCF(2,2)/def2-TZVP optimized geometries. C_b_ and C_m_ in **1**–**3** and **5** denote bridgehead and methylene carbon atoms, respectively.

Key interatomic distances from the geometries of [1.1.1]propellane **1**, bicyclo[1.1.0]butane **2**, bicyclo[1.1.1]pentane **3**, tetrahedrane **4**, and cyclopropane **5** that were used in the off-nucleus isotropic magnetic shielding calculations are shown in Figure 1.

As is to be expected from previous work,^6^ B3LYP underestimates the C_b_–C_b_ distance in **1,** whereas CASSCF(2,2) gets it about right; a similar situation is observed in **2**. The C_b_–C_b_ distances from our B3LYP-D3(BJ)/def2-TZVP and CASSCF(2,2)/def2-TZVP optimized geometries of **1** are in excellent agreement with those obtained at the B3LYP/def2-QZVPP and CASSCF(2,2)/def2-QZVPP levels, respectively;^6^ very close agreement is also observed between the B3LYP-D3(BJ)/def2-TZVP and B3LYP/cc-pVTZ^34^ optimized geometries of **3** and its experimental geometry. These observations indicate that the use of larger basis sets and the addition of dispersion corrections to B3LYP have very minor effects on the optimized geometries of **1** and **3**.

**Figure 2:**
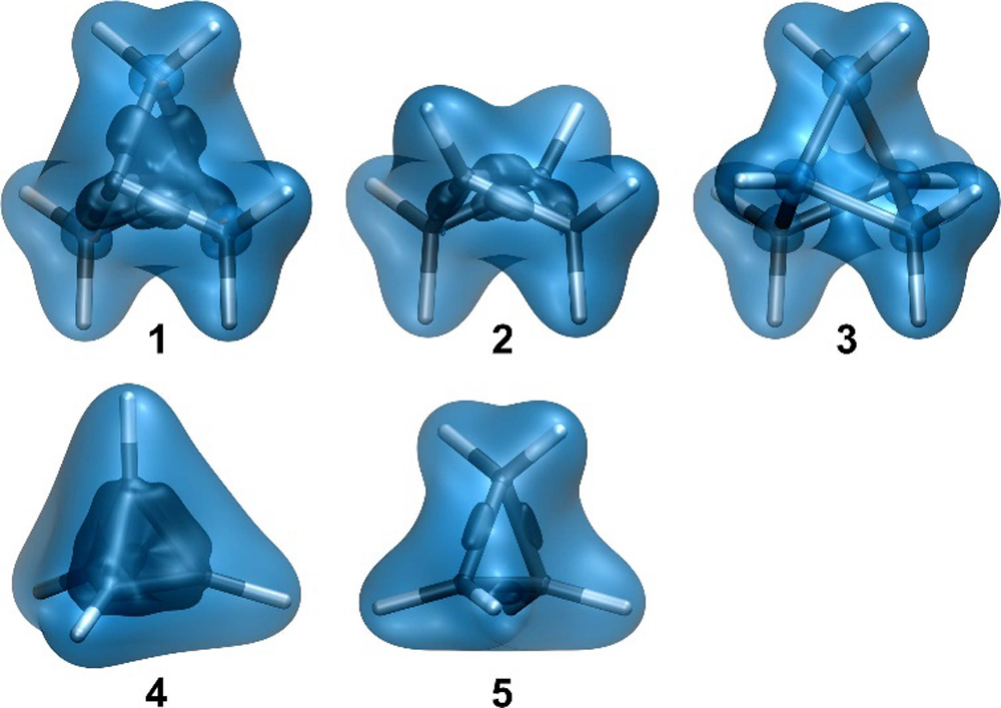
Isotropic shielding isosurfaces for **1**–**5** at σ_iso_(**r**) = ±16 ppm (positive/negative isovalues in blue/orange) and σ_iso_(**r**) = 50 ppm (darker) [B3LYP-GIAO/6-311++G(d,p)//B3LYP-D3(BJ)/def2-TZVP].

**Figure 3:**
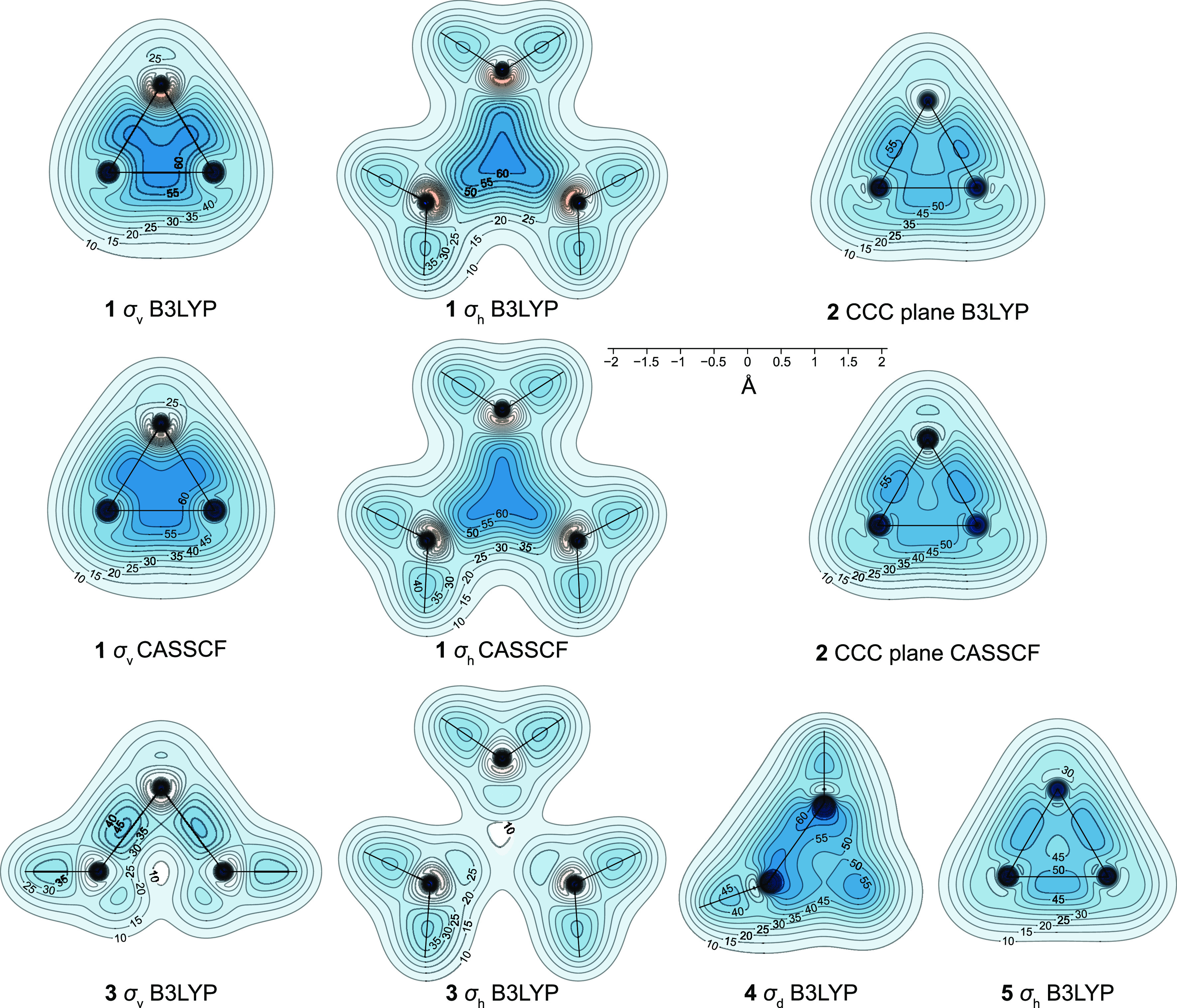
Isotropic shielding contour plots in the σ_v_, σ_h_, or d σ_d_ symmetry planes of **1**, **3**–**6** and in one of the CCC planes of **2**, from calculations at the B3LYP-GIAO/6-311++G(d,p)//B3LYP-D3(BJ)/def2-TZVP and CASSCF(2,2)-GIAO/6-311++G(d,p)//CASSCF(2,2)/def2-TZVP levels. Lines show bonds in the plotting plane. σ_iso_(**r**) range between ca. −20 and 180 ppm, red/orange (deshielded) to blue (shielded).

The changes in isotropic shielding around **1**–**5** from data computed at the B3LYP level are illustrated in Figure 2. The CASSCF(2,2) isosurfaces for **1** and **2** are visually very similar to the B3LYP ones and so they are not shown here separately (but these isosurfaces can be examined using the corresponding GAUSSIAN cube files in the Supporting Information). We observe that all C–C and C–H bonds in **1**–**5** are well-shielded, in a fashion similar to what has been observed in off-nucleus shielding calculations on other molecules.^18,19^ Due to the relatively small separations between the various C–C bonds in **1**–**5**, the shielded regions around these bonds show a tendency to merge together within the σ_iso_(**r**) = 16 ppm isosurface. There is, however, a “shielding hole” of σ_iso_(**r**) < 10 ppm near the center of **3** and there are regions of decreased shielding near the centers of **4** and **5**. (These features are easier to observe in the respective contour plots in Figure 3, as discussed later.)

Shielding within the darker regions just outside each of the C–C bonds in cyclopropane **5** exceeds 50 ppm. The positioning of these strongly shielded regions supports the Coulson–Moffitt model of bonding in this molecule,^35^ in which three bent C–C bonds are formed from six equivalent HAOs that overlap in pairs just outside the triangle formed by the three carbon atoms, and is in agreement with the results of spin-coupled generalized VB (SCGVB) calculations.^36^ The lower shielding in the ring’s interior makes less likely the alternative Walsh model^37^ in which the overlap of three carbon sp^2^ HAOs pointing toward the center of the ring gives rise to a two-electron three-center bond.

The strongly shielded region encompassing the middle parts of the wing C–C bonds and most of the interior of the [1.1.1]propellane cage in **1** has no counterpart in **3**, where shielding does not reach 50 ppm anywhere in the vicinity of its longer and more widely spaced wing C–C bonds. In contrast to the situation in **5**, we do not observe any signs of repulsive interactions between the wing C–C bonds; in fact, the strongly shielded central region in **1** suggests that most of the shielding over each of these bonds remains inside the cage and contributes to this strongly shielded central region. Connected regions inside which σ_iso_(**r**) exceeds 50 ppm are also observed in **2** and **4**. While that in **2** is smaller than the corresponding region in **1**, the increased shielding “grips” the wing C–C bonds in a very similar manner. The close proximity of the six C–C bonds making up the very compact tetrahedrane cage **4** leads to the appearance of sizable-connected strongly shielded regions over these bonds. (The respective contour plot in Figure 3 shows that each of these regions bends out of the cage and that shielding decreases toward the cage center.)

The contour plots shown in Figure 3 provide more detailed information about the spatial variations in the isotropic shielding around **1**–**5**. Whereas the shielding contours outline the C–C bonds in **2**–**5** reasonably well, it turns out not to be at all straightforward to think of a way of separating the strongly shielded central region in **1** into contributions associated with individual bonds. On the other hand, there is not even a trace of a shielded central region in **3**. Indeed, the contour plots in the σ_h_ and σ_v_ symmetry planes of **3** show that shielding near the center of this molecule goes down to under 10 ppm. We note that the σ_h_ contour plot for **5** lends further support to the bent C–C bonds model of this molecule. Different extents of C–C bond outward “bending” are also observed in **2**, **3**, and **4**, but not in **1**. As can be seen from the contour plots obtained at the CASSCF(2,2) level, the size of the strongly shielded central region in **1** turns out to be larger at this level, in spite of the longer distance between the bridgehead carbon atoms; increased shielding is also observed within the three-membered ring in the CCC plane in **2**.

The methylene carbon atoms in **1** are surrounded by small ovoid deshielded regions inside which σ_iso_(**r**) becomes negative. (These regions are more obvious in the contour plots in Figure 3 and are more pronounced at the B3LYP level). Similar deshielded “halos” around sp^2^ and sp hybridized carbon atoms and other sp^2^ hybridized first main row atoms have been observed previously in conjugated rings,^21,22,38,39^ as well as in open-chain and conjugated molecules such as ethene, ethyne, and *s*-*trans*-1,3-butadiene.^18,19^ These “halos” have been attributed to a specific type of π electron behavior that is a characteristic of some sp^2^ and sp hybridized first main row atoms and that is different from traditional ring currents.^21^ The occurrence of such “halos” around the methylene carbon atoms in **1** suggests a hybridization state that is in-between sp^2^ and sp^3^. There are no such “halos” around the bridgehead carbon atoms in **1** and **2**, or any of the carbon atoms in **4** and **5**; accordingly, the hybridization states of all of these carbon atoms are expected to be close to sp^3^. The surroundings of the methylene carbon atoms in **2** and of all the carbon atoms in **3** do show some deshielding, less pronounced than that around the methylene carbon atoms in **1**, but still clearly noticeable, even in Figure 2 (see the almost spherical parts of the isotropic shielding isosurface at 16 ppm surrounding all carbon atoms in **3**). This does come as a surprise and while one interpretation could be that the carbon atoms in **3** also have hybridization states intermediate between sp^2^ and sp^3^, some of this deshielding could also be due to the longer wing C–C bonds. Very close to carbon nuclei, the isotropic shielding is always positive and it increases sharply, as has been shown in detail for the sp hybridized carbon atoms in C_2_H_2_.^19^

One notable feature of the isotropic shielding variations around **1** is the absence of shielded regions close to the bridgehead carbon atoms that would be expected to arise from “exo” sp^3^-like HAOs on these atoms pointing outward of the [1.1.1]propellane cage. While at the B3LYP level, this could be partially attributed to the use of doubly occupied Kohn–Sham orbitals, the analogous CASSCF(2,2) isotropic shielding results indicate that this is indeed a feature of the shielding distribution in this molecule: We observe no increased shielding resulting from the two outward-directed orthogonal localized active space CASSCF(2,2) orbitals reported by Duarte and co-workers,^6^ the shapes of which closely resemble “exo” sp^3^-like HAOs. Moreover, the shielding picture around the bridgehead carbon atoms in **1** is markedly different from that for C_2_, in which there are sizable regions of increased shielding outward of the C–C bond that are consistent with the shielding actions of two “exo” sp HAOs.^19^ The shielding variations around **1** strongly suggest that the shielding activities of any HAOs on the bridgehead carbon atoms which are not involved in the C_b_–C_m_ bonds are almost entirely contained within the [1.1.1]propellane cage.

**Figure 4:**
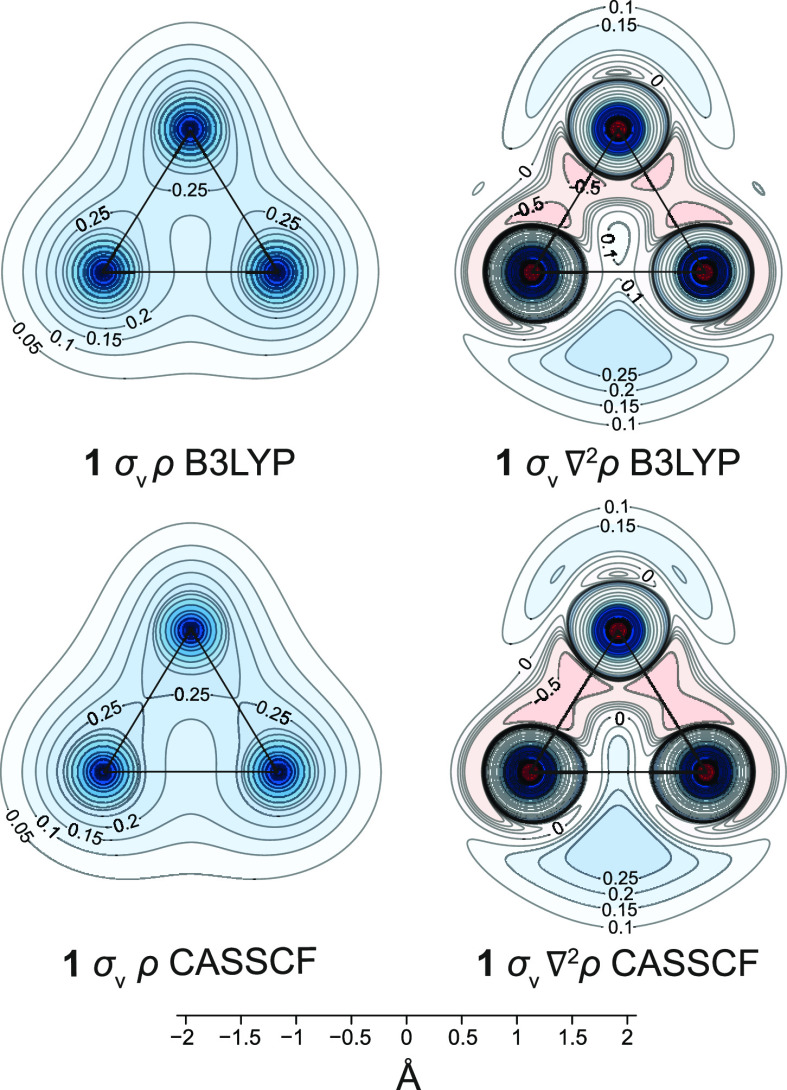
Total electron density (ρ) and Laplacian of the total electron density (∇^2^ρ) contour plots in the σ_v_ symmetry plane of **1**, from calculations at the B3LYP/6-311++G(d,p)//B3LYP-D3(BJ)/def2-TZVP and CASSCF(2,2)/6-311++G(d,p)//CASSCF(2,2)/def2-TZVP levels. Lines show bonds in the plotting plane. ρ range between 0 and 75 a.u. (ρ, blue), ∇^2^ρ range between ca. −10^5^ and 300 a.u. (∇^2^ρ, red to blue).

The shielding picture outside the [1.1.1]propellane cage is consistent with the total electron density distribution in that region of space. The B3LYP and CASSCF(2,2) total electron density (ρ) contour plots shown in Figure 4 are reasonably similar. A notable feature of both of these plots is the lower electron density inside the [1.1.1]propellane cage, along the C_b_–C_b_ direction, when compared to that along the C_b_–C_m_ bonds. This is in agreement with the experimental and B3LYP/6-311G* static deformation densities for **1**.^40^ While highlighting clearly the C_b_–C_m_ bonds, the Laplacian of the total electron density (∇^2^ρ), at either level of theory used here, does not show a C_b_–C_b_ interaction of the same type (see Figure 4).

One argument that has often been used in support of the existence of a C_b_–C_b_ bond is associated with the presence of a bond critical point (bcp) at the midpoint of the line connecting the two bridgehead carbon atoms.^40,41^ However, while at the Hartree–Fock level (HF/6-31G*), the Laplacian at this bcp was found to be negative (−0.109 a.u.),^41^ which is suggestive of some covalent bond character, subsequent evaluations^40^ at the B3LYP/6-311G* level, and from experimental electron densities produced positive ∇^2^ρ values of 0.083 and 0.427 a.u., respectively, which are more in line with a noncovalent interaction. Similarly, our B3LYP and CASSCF(2,2) values of ∇^2^ρ at the midpoint of the C_b_–C_b_ line, extracted from the data used to construct Figure 4, are 0.093 and 0.179 a.u., respectively, again suggestive of a noncovalent interaction. While still smaller than the experimental ∇^2^ρ value, the CASSCF(2,2) result is a significant improvement, in the right direction, over that obtained at the B3LYP level.

It is important to note that the ρ and ∇^2^ρ contour plots in Figure 4 do not display features that could be used to account for the presence of a strongly shielded region within the interior of the [1.1.1]propellane cage (see Figures 2 and 3). Still, by analogy to the increased shielding over chemical bonds observed in other molecules, it is logical to assume that this shielded region is linked to the existence of some form of bonding interaction. The appearance of this shielded region can be associated, in part, with the overlaps of the shielded regions over the six closely spaced C_b_–C_m_ bonds and, indeed, the contour plots in Figure 3 show that the overlaps of the shielded regions over neighboring C–C bonds do increase in the sequence cyclopropane **5** (one three-membered carbon ring), bicyclo[1.1.0]butane **2** (two fused three-membered carbon rings), [1.1.1]propellane **1** (three three-membered carbon rings fused over the link between the bridgehead carbon atoms). On the other hand, such overlaps should be more pronounced in the even smaller interior of tetrahedrane **4** and yet the shielding decreases toward the center of the cage.

**Figure 5:**
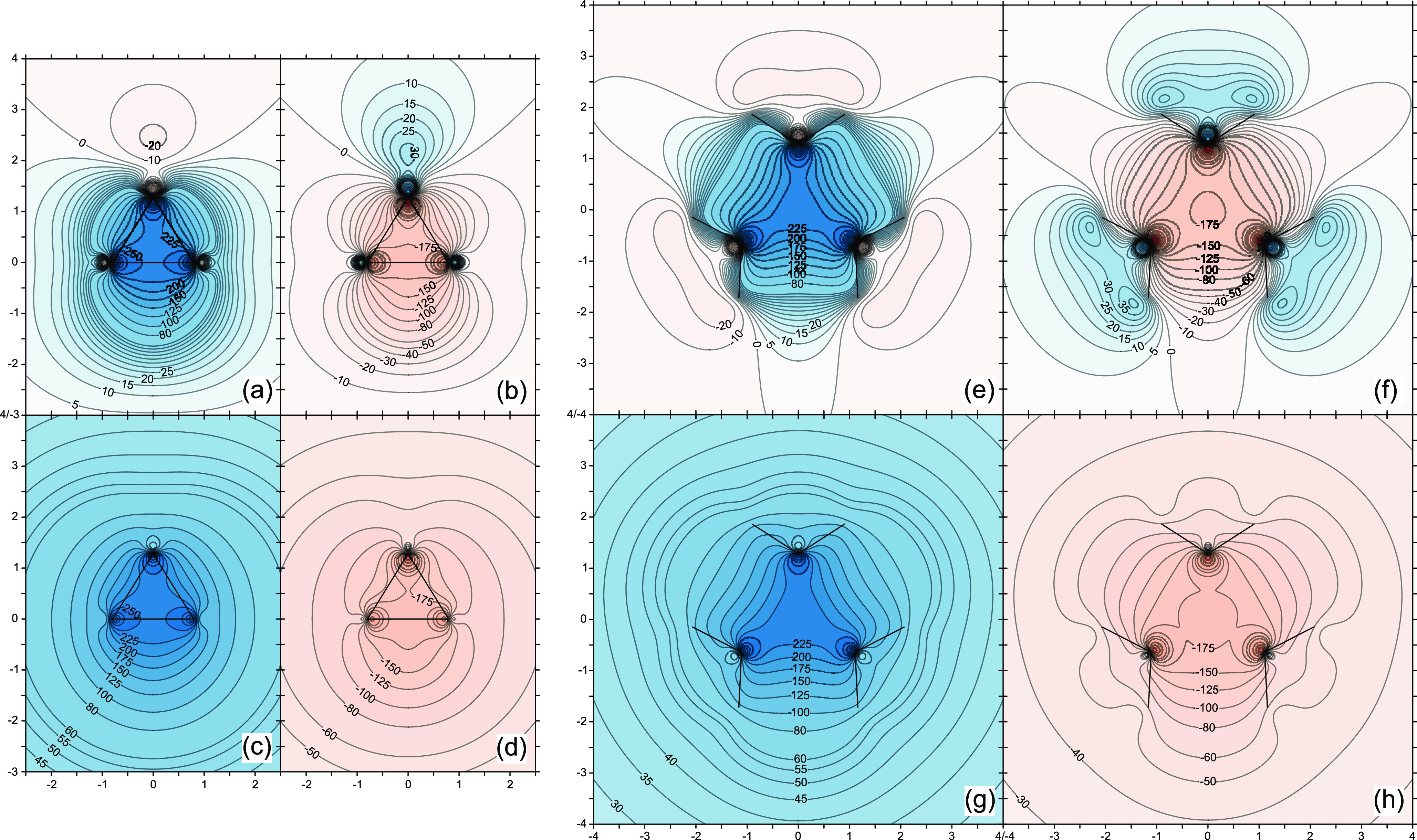
Contour plots of the diamagnetic (a, c, e, g) and paramagnetic (b, d, f, h) contributions to the isotropic shielding in **1** in the σ_v_ (a–d) and σ_h_ (e–h) symmetry planes from calculations at the CASSCF(2,2)-GIAO/6-311++G(d,p)//CASSCF(2,2)/def2-TZVP level. (a, b, e, f) were calculated with gauge origin at the center of mass; (c, d, g, h) were calculated with individual gauge origins at **r** for each **σ**(**r**). Lines show bonds in the plotting plane. The σ_iso_^d^(**r**) and σ_iso_^p^(**r**) ranges are between ca. −600 and 600 ppm, red/orange (deshielded) to blue (shielded), axes in Å.

Further insights into the nature of the strongly shielded central region in **1** can be obtained by examining the diamagnetic and paramagnetic contributions to the off-nucleus isotropic shielding. As the CASSCF(2,2) level provides a more reliable picture of the electronic structure of **1**, the data for the σ_iso_^d^(**r**) and σ_iso_^p^(**r**) contour plots in Figure 5 come from calculations at this level rather than B3LYP. The diamagnetic and paramagnetic contributions depend on the choice of the gauge origin and there are two possible choices which ensure that these contributions reflect the full symmetry of **1**. The first of these is to go along with the standard single gauge origin at the center of mass, and the alternative is to use an individual gauge origin at **r** for each **σ**(**r**). We have carried out σ_iso_^d^(**r**) and σ_iso_^p^(**r**) calculations with each of these choices. In both cases, the calculations were performed using the natural connection.

As can be seen in Figure 5, the behavior of each of the diamagnetic and paramagnetic contributions inside the [1.1.1]propellane cage does not change much on switching from a single gauge origin at the center of mass to individual gauge origins at **r** for each **σ**(**r**): for either choice, σ_iso_^d^(**r**) and σ_iso_^p^(**r**) shield and deshield the interior of the cage, respectively. More pronounced differences are observed outside the [1.1.1]propellane cage: With a single gauge origin at the center of mass, σ_iso_^d^(**r**) and σ_iso_^p^(**r**) shield and deshield, respectively, the immediate surroundings of the cage, change sign in certain regions of space outside the cage, and the magnitudes of both contributions decrease quickly with increasing distance from the center of the cage. On the other hand, with individual gauge origins at **r** for each **σ**(**r**), σ_iso_^d^(**r**) and σ_iso_^p^(**r**) turn out to be uniformly positive and negative, respectively, and the magnitude of each of these contributions decreases slower with increasing distance from the center of the cage.

Interestingly, for either choice of gauge origin, inside the cage, the variations in both the shielding from σ_iso_^d^(**r**) and the deshielding from σ_iso_^p^(**r**) suggest that these could arise through the interactions between three Walsh-model-like sp^2^ HAOs on the methylene carbon atoms, pointing toward the center of the cage and two sp^3^ HAOs on the bridgehead carbon atoms. Of course, when added together, the σ_iso_^d^(**r**) and σ_iso_^p^(**r**) contour plots for either choice of gauge origin in Figure 5 reproduce the corresponding σ_iso_(**r**) contour plots in Figure 3, with shielding prevailing almost everywhere, but the details that can be associated with HAOs are no longer obvious. Similarly to the σ_iso_(**r**) contour plots in the σ_v_ plane (Figure 3), the corresponding σ_iso_^d^(**r**) and σ_iso_^p^(**r**) contour plots do not show significant shielding or deshielding outside the cage that could result from the two outward-directed orthogonal localized active space CASSCF(2,2) orbitals on the bridgehead carbon atoms. The observation that the diamagnetic contribution to the isotropic shielding, which depends on the electron density, behaves differently from the total electron density (compare the σ_v_ contour plots in Figures 4 and 5) lends further support to the argument that the shielding picture inside the [1.1.1]propellane cage would be very difficult to explain by examining only the total electron density and/or its Laplacian. The bonding interaction associated with the increased shielding within the [1.1.1]propellane cage can be overlooked by approaches that partition the total electron density into contributions from orbitals and/or VB structures, or that examine its Laplacian. Overall, our conclusion is that the shielding picture inside the [1.1.1]propellane cage suggests the existence of a bonding interaction which cannot be separated, in a straightforward fashion, into contributions from individual carbon–carbon bonds. The bridgehead carbon atoms are fully engaged in this bonding interaction and, as mentioned above, the shielding activity of the “exo” sp^3^-like HAOs is almost entirely contained within the cage.

While the accurate reproduction of the experimentally measured isotropic shieldings and chemical shifts in **1**–**5** is not amongst the aims of this paper, it is interesting to examine the extent to which our B3LYP-GIAO and CASSCF-GIAO results obtained using the 6-311++G(d,p) basis set (see Table 1) agree with experimental data and with other theoretical estimates. A detailed comparison between a number of theoretical estimates and experimental measurements of the isotropic shieldings of the nuclei in **1** and **3** has been carried out by Pecul and co-workers.^42^ Despite the use of a slightly different geometry, our CASSCF(2,2) σ_iso_(^13^C_b_, **1**) value of 200.6 ppm is very close to their RAS-II/IGLO-III (restricted active space SCF with GIAOs) value of 199.2 ppm which, in turn, is in excellent agreement with the experiment. There is a larger difference between the CASSCF(2,2) and RAS-II/IGLO-III σ_iso_(^13^C_m_, **1**) values of 129.0 and 122.1 ppm, respectively; this can be attributed to the much smaller active space in the CASSCF(2,2) wavefunction, which is limited to just two orbitals on the bridgehead carbon atoms. Similar considerations apply to the difference between the CASSCF(2,2) and RAS-II/IGLO-III σ_iso_(^1^H_m_, **1**) values of 30.9 and 30.1 ppm. The B3LYP-GIAO σ_iso_(^13^C) values for **1** obtained using the IGLO-III basis (177.9/100.0 ppm)^42^ turned out to be lower than the experimental measurements. Our B3LYP σ_iso_(^13^C) values are higher but still below the experimental values; B3LYP performs reasonably well for σ_iso_(^1^H_m_) in **1**. Additionally, some of the differences between our gas-phase CASSCF(2,2)-GIAO σ_iso_(^13^C) values for **1** and **2**, and B3LYP-GIAO σ_iso_(^13^C) values for **1**, **2**, and **5** are very close to the differences between the corresponding liquid NMR shielding constants, taken with negative signs.^43^ CASSCF(2,2)-GIAO and B3LYP-GIAO calculations estimate the σ_iso_(^13^C_b_, **1**)−σ_iso_(^13^C_m_, **1**) difference as 71.6 and 73.5 ppm, respectively (see Table 1), with the latter being very close to the liquid NMR value of 73.2 ppm; the CASSCF(2,2)-GIAO value is less accurate because of the small active space. Our CASSCF(2,2)-GIAO and B3LYP-GIAO σ_iso_(^13^C_b_, **2**)−σ_iso_(^13^C_b_, **1**) differences are 2.9 and 3.0 ppm, respectively, against a liquid NMR difference of 4 ppm, and the CASSCF(2,2)-GIAO and B3LYP-GIAO σ_iso_(^13^C_m_, **2**)−σ_iso_(^13^C_m_, **1**) differences are 40.5 and 43.3 ppm, respectively, against a liquid NMR difference of 41.2 ppm. At the B3LYP-GIAO level, σ_iso_(^13^C_b_, **1**)−σ_iso_(^13^C, **5**) = −3.4 ppm (from Table 1) is in excellent agreement with the liquid NMR difference of −3.8 ppm. All in all, as is to be expected from the literature,^44^ shieldings calculated at the B3LYP-GIAO/6-311++G(d,p) level correlate well with experimental NMR data for molecules such as those included in Table 1, and it turns out that the CASSCF(2,2)-GIAO/6–311++G(d,p) level also performs reasonably well.

**Table 1:** Isotropic Shieldings of All Nuclei in [1.1.1]Propellane **1**, Bicyclo[1.1.0]butane **2**, Bicyclo[1.1.1]pentane **3**, Tetrahedrane **4**, Cyclopropane **5**, Ethane, and Ethene (in ppm)^a^

molecule	method	σ_iso_(^13^C)	σ_iso_(^1^H)
**1**	B3LYP	183.1/109.6	30.3
	CASSCF	200.6/129.0	30.9
**2**	B3LYP	186.1/152.9	30.8/31.6/30.7
	CASSCF	203.5/169.5	31.2/32.1/31.3
**3**	B3LYP	147.5/131.0	29.5/30.2
**4**	B3LYP	211.8	29.1
**5**	B3LYP	186.5	32.1
ethane	B3LYP	176.3	31.2
ethene	B3LYP	53.8	26.3

a Gas-phase B3LYP-GIAO and CASSCF(2,2)-GIAO calculations in the 6-311++G(d,p) basis set at optimized or experimental geometries (for details, see text).^13^C_b_/^13^C_m_ values for **1**–**3**,^1^H_b_/^1^H_m_(axial)/^1^H_m_(equatorial) values for **2**,^1^H_b_/^1^H_m_ values for **3**.

Looking again at the B3LYP-GIAO results in Table 1, we observe that the σ_iso_(^13^C_b_, **1**) and σ_iso_(^13^C, **5**) values are close to, but higher than, the carbon isotropic shielding in ethane, a molecule with sp^3^ hybridized carbon atoms. This strengthens the argument made on the basis of the isotropic shielding plots for **1** and **5** (see Figure 3) that the hybridization states of the bridgehead carbon atoms in **1** and all of the carbon atoms in **5** should be close to sp^3^. On the other hand, the σ_iso_(^13^C_m_, **1**) value lies between the carbon isotropic shieldings in ethane and ethene, but it is closer to that in ethene, consistent with the observation of the deshielded “halos” around the methylene carbon atoms in **1** (see Figure 3). The σ_iso_(^13^C_b_, **2**) and σ_iso_(^13^C_m_, **2**) values also lie between the carbon isotropic shieldings in ethane and ethene, but they are closer to that in ethane, consistent with the less pronounced deshielded “halos” around the respective carbon atoms that are observed in Figure 3.

The proton isotropic shieldings in **1** are higher than those in ethene and the σ_iso_(^1^H_m_) values decrease with the additions of the second and third fused three-membered rings in the sequence **5**, **2**, **1**. Even so, the σ_iso_(^1^H_m_) values in **1** and **2** remain closer to the proton isotropic shielding in ethane than to that in ethene. This is most likely due to the absence of π electron systems in **1** and **2** and should not be interpreted as an indication that the hybridization states of the methylene carbon atoms in these molecules are closer to sp^3^ than to sp^2^.

## Conclusions

The off-nucleus isotropic magnetic shielding and its diamagnetic and paramagnetic contributions studied as functions of position in the space surrounding [1.1.1]propellane show that the shielding activity of the two “exo” sp^3^-like HAOs on the bridgehead carbon atoms (say, in the form of localized CASSCF orbitals) used to support the idea of an inverted bond between these carbons is almost entirely contained within the [1.1.1]propellane cage. We observe a strongly shielded central region within this cage that encloses most of its interior and extends over the middle parts of the wing C–C bonds. The diamagnetic and paramagnetic contributions to shielding within this region could be thought to arise through the interactions between three Walsh-model-like sp^2^ HAOs on the methylene carbon atoms, pointing toward the center of the cage and two sp^3^ HAOs on the bridgehead carbon atoms. The size and intensity of the central shielded region suggest the existence of a reasonably strong bonding interaction which cannot be separated, in a straightforward fashion, into contributions from individual carbon–carbon bonds; the bridgehead carbon atoms are fully engaged in this bonding interaction. Outside the cage, our results show no significant shielding next to the bridgehead carbon atoms. The comparison of the results of B3LYP and CASSCF(2,2) calculations on [1.1.1]propellane to those for other molecules involving three-membered carbon rings, namely, bicyclo[1.1.0]butane, bicyclo[1.1.1]pentane, tetrahedrane, and cyclopropane, suggests that this interaction inside the [1.1.1]propellane cage is predominantly covalent in nature.

Of course, the electronic structure of [1.1.1]propellane could be interpreted in more than one way, for example, using localized MOs or different VB approaches, placing more or less emphasis on orbital shapes, overlaps, and ionic structures. A discussion of the pros and cons of such alternative interpretations of [1.1.1]propellane might not seem so dissimilar to comparisons between the alternative σ-π and bent-bond descriptions of multiple carbon–carbon bonds.^45−47^ However, the magnetic shielding interpretation of bonding in this molecule would not change as it does not require the use of a specific wavefunction—as we have demonstrated, the analyses of B3LYP and CASSCF(2,2) isotropic shielding contour plots lead to essentially the same conclusions.

Our analysis of the off-nucleus isotropic magnetic shielding results for [1.1.1]propellane does of course challenge simple notions that the bonding in almost any molecule can be described by drawing lines connecting atoms—according to our results, the bonding interactions in tight spaces, such as in the [1.1.1]propellane cage, can be rather more complicated.

## Supporting Information Available

Additional computational details and optimized geometries for [1.1.1]propellane, bicyclo[1.1.0]butane, bicyclo[1.1.1]pentane, and tetrahedrane (PDF)
Gaussian cube files with isotropic shielding values for [1.1.1]propellane, bicyclo[1.1.0]butane, bicyclo[1.1.1]pentane, tetrahedrane, and cyclopropane (ZIP).
High resolution versions of all figures (ZIP)
