# Supplementary material for: Magnetic Shielding Analysis of Bonding in [1.1.1]Propellane
Source: J Phys Chem A. 2023 Jan 19;127(4):861–9. doi: 10.1021/acs.jpca.2c06450 (PMC9900594; doi:10.1021/acs.jpca.2c06450)
Supplement: Supplementary file 1 — jp2c06450_si_001.pdf [file jp2c06450_si_001.pdf]

Supporting Information

Magnetic Shielding Analysis of Bonding in [1.1.1]Propellane

Peter B. Karadakov,*† Ben Stewart† and David L. Cooper‡

†Department of Chemistry, University of York, Heslington, York YO10 5DD, U.K.
‡Department of Chemistry, University of Liverpool, Liverpool L69 7ZD, U.K.

*E-mail: peter.karadakov@york.ac.uk

1. Gaussian Cube Files with Isotropic Shielding Values

A ZIP archive of Gaussian cube files with isotropic shielding values for [1.1.1]propellane, bicyclo[1.1.0]butane, bicyclo[1.1.1]pentane, tetrahedrane and cyclopropane obtained at the B3LYP-GIAO/6-311++G(d,p)//B3LYP-D3(BJ)/def2-TZVP and CASSCF(2,2)-GIAO/6-311++G(d,p)//CASSCF(2,2)/def2-TZVP ([1.1.1]propellane and bicyclo[1.1.0]butane only) levels is available as a separate download.

2. Additional Computational Details

All GAUSSIAN B3LYP-D3(BJ)/def2-TZVP geometry optimizations and vibrational frequencies calculations for [1.1.1]propellane, bicyclo[1.1.0]butane, bicyclo[1.1.1]pentane, and tetrahedrane reported in this paper were carried out using the options "Opt(VeryTight) EmpiricalDispersion=GD3BJ Int(Grid=SuperFine)".

All GAUSSIAN B3LYP-GIAO/6-311++G(d,p) NMR shielding tensor calculations for [1.1.1]propellane, bicyclo[1.1.0]butane, bicyclo[1.1.1]pentane, tetrahedrane, cyclopropane, ethane and ethene reported in this paper were carried out using the options "SCF(Tight) CPHF(Separate) Int(Grid=SuperFine)".

The GAUSSIAN CASSCF(2,2)/def2-TZVP geometry optimization and vibrational frequencies calculations on [1.1.1]propellane and bicyclo[1.1.0]butane were run using the options "Opt(VeryTight) Freq(Numer)" as the attempt to use analytical frequencies failed with a program error.

The DALTON CASSCF(2,2)-GIAO/6-311++G(d,p) NMR shielding tensor calculations for [1.1.1]propellane and bicyclo[1.1.0]butane were run without changes to the default program options.

3. Optimized Geometries and Other Computational Data

All coordinates are in Å.

B3LYP-D3(BJ)/def2-TZVP total energy E, lowest vibrational frequency ν and optimized geometry of [1.1.1]propellane (D_{3h} symmetry).

$E = -194.092860$ Ha; $\nu = 533.7\ \mathrm{cm}^{-1}$ (E′).

C	0.000000	0.000000	0.783342
C	0.000000	0.000000	-0.783342
C	0.000000	1.296227	0.000000
C	1.122565	-0.648113	0.000000
C	-1.122565	-0.648113	0.000000
H	-1.168191	-1.729863	0.000000
H	-2.082201	-0.146752	0.000000
H	1.168191	-1.729863	0.000000
H	2.082201	-0.146752	0.000000
H	-0.914010	1.876615	0.000000
H	0.914010	1.876615	0.000000

CASSCF(2,2)/def2-TZVP total energy E, lowest vibrational frequency ν and optimized geometry of [1.1.1]propellane (D_{3h} symmetry).

E = –192.788932 Ha; ν = 592.5 cm^{-1} (E′).

C	0.000000	-0.000000	-0.795168
C	0.000000	-0.000000	0.795168
C	0.000000	1.281289	0.000000
C	1.109629	-0.640645	0.000000
C	-1.109629	-0.640645	0.000000
H	-1.161553	-1.714744	0.000000
H	-2.065789	-0.148562	0.000000
H	1.161553	-1.714744	0.000000
H	2.065789	-0.148562	0.000000
H	-0.904236	1.863307	0.000000
H	0.904236	1.863307	0.000000

B3LYP-D3(BJ)/def2-TZVP total energy E, lowest vibrational frequency ν and optimized geometry of bicyclo[1.1.0]butane (C_{2v} symmetry).

E = – 156.018565 Ha; ν = 419.0 cm^{-1} (A_1).

C	0.742485	0.000000	0.311174
C	0.000000	1.133920	-0.318837
H	0.000000	1.232200	-1.403856
H	-0.000000	2.078181	0.214936
C	-0.742485	-0.000000	0.311174
C	0.000000	-1.133920	-0.318837
H	0.000000	-1.232200	-1.403856
H	0.000000	-2.078181	0.214936
H	1.424473	0.000000	1.143752
H	-1.424473	-0.000000	1.143752

CASSCF(2,2)/def2-TZVP total energy E, lowest vibrational frequency ν and optimized geometry of bicyclo[1.1.0]butane (C_{2v} symmetry).

E = – 154.944234 Ha; ν = 470.2 cm^{-1} (A_1).

C	-0.748784	0.000000	-0.314424
C	0.748784	-0.000000	-0.314424
H	0.000000	1.124322	0.308283

H	-0.000000	-1.124322	0.308283
C	0.000000	1.226756	1.384276
C	-0.000000	-1.226756	1.384276
H	0.000000	2.064128	-0.218396
H	-0.000000	-2.064128	-0.218396
H	-1.439139	0.000000	-1.129035
H	1.439139	-0.000000	-1.129035

B3LYP-D3(BJ)/def2-TZVP total energy E, lowest vibrational frequency ν and optimized geometry of bicyclo[1.1.1]pentane (D_{3h} symmetry).

E = –195.347529 Ha; ν = 541.8 cm^{-1} (E′).

C	0.000000	0.000000	0.937484
C	0.000000	-0.000000	-0.937484
C	0.000000	1.238434	0.000000
C	1.072516	-0.619217	0.000000
C	-1.072516	-0.619217	0.000000
H	-1.151884	-1.707069	0.000000
H	-2.054307	-0.144026	0.000000
H	1.151884	-1.707069	0.000000
H	2.054307	-0.144026	0.000000
H	-0.902423	1.851095	0.000000
H	0.902423	1.851095	0.000000
H	0.000000	0.000000	2.026356
H	0.000000	0.000000	-2.026356

B3LYP-D3(BJ)/def2-TZVP total energy E, lowest vibrational frequency ν and optimized geometry of tetrahedrane (T_d symmetry).

E = –154.706283 Ha; ν = 572.6 cm^{-1} (E).

C	0.521091	0.521091	0.521091
C	-0.521091	-0.521091	0.521091
C	0.521091	-0.521091	-0.521091
C	-0.521091	0.521091	-0.521091
H	1.138027	1.138027	1.138027
H	-1.138027	-1.138027	1.138027
H	-1.138027	1.138027	-1.138027
H	1.138027	-1.138027	-1.138027
