# Supplementary figures and images for: Magnetic Shielding Analysis of Bonding in [1.1.1]Propellane
Source: J Phys Chem A. 2023 Jan 19;127(4):861–9. doi: 10.1021/acs.jpca.2c06450 (PMC9900594; doi:10.1021/acs.jpca.2c06450)

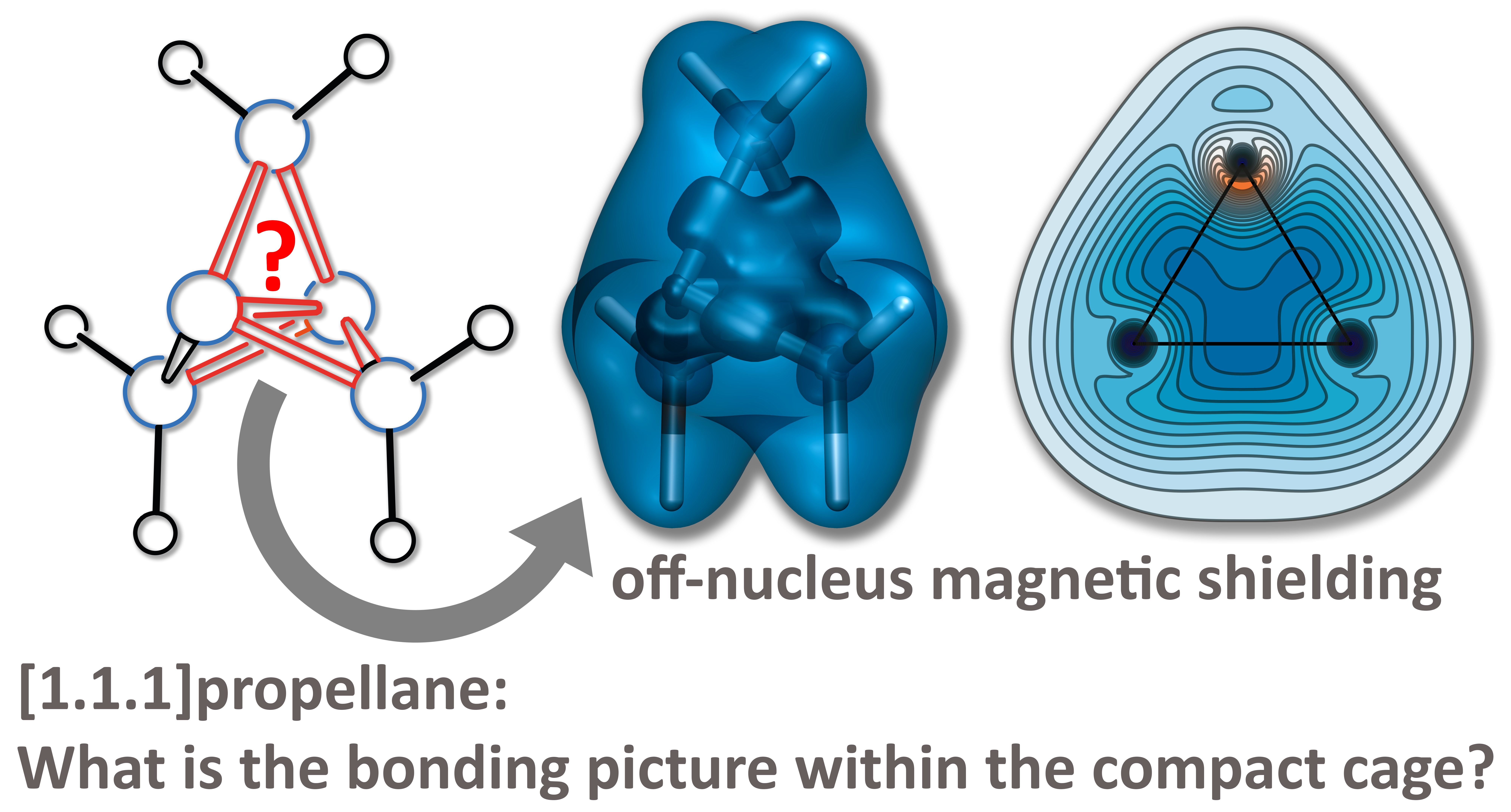

Supplement: Supplementary file 3 — jp2c06450_si_003.zip [file jp2c06450_si_003.zip › 111propellane-toc.tif]
